# Identification of the Sex of Pre-implantation Mouse Embryos Using a Marked Y Chromosome and CRISPR/Cas9

**DOI:** 10.1038/s41598-019-50731-x

**Published:** 2019-10-04

**Authors:** Xiuling Zhao, Wei Wei, Hong Pan, Junyu Nie, Dongrong Chen, Pengfei Zhang, Fumei Chen, Qiang Fu, Erwei Zuo, Yangqing Lu, Ming Zhang

**Affiliations:** 1 State Key Laboratory for Conservation and Utilization of Subtropical Agro-Bioresources, Animal Reproduction Institute, Guangxi University, Nanning, 530004 Guangxi P.R. China; 2 Shenzhen Hengsheng Hospital, Shenzhen, 518101 P.R. China; 3 Center for Research in Animal Genomics, Agricultural Genome Institute at Shenzhen, Chinese Academy of Agricultural Sciences, Shenzhen, 518124 P.R. China

**Keywords:** Genetic engineering, Molecular biology

## Abstract

Although numerous attempts have been made to alter the sex ratio of the progeny of mammals, the limitations of current technologies have prevented their widespread use in farm animals. The presence or absence of a Y chromosome determines whether a mammalian embryo develops as a male or female, and non-invasive genetic reporters such as fluorescence protein markers have been intensively applied in a variety of fields of research. To develop a non-invasive and instantaneous method for advance determination of the sex of embryos, we developed a Y chromosome-linked *eGFP* mouse line that stably expresses green fluorescent protein under the control of the CAG promoter. The development of the CRISPR/Cas9 system has made it easy to deliver an exogenous gene to a specific locus of a genome, and linking a tracer to the Y chromosome has simplified the process of predicting the sex of embryos collected by mating a Y-Chr-eGFP transgenic male with a wild-type female. XY embryos appeared green, under a fluorescence microscope, and XX embryos did not. Y chromosome-linked genes were amplified by nested PCR to further confirm the accuracy of this method, and the simultaneous transplantation of green and non-green embryos into foster mothers indicated that 100% accuracy was achieved by this method. Thus, the Y-Chr-eGFP mouse line provides an expeditious and accurate approach for sexing pre-implantation embryos and can be efficiently used for the pre-selection of sex.

## Introduction

The ability to produce offspring of a desired sex is significant for livestock production, and several approaches have been developed to determine the sex of embryos prior to their transfer in mammals. Although Veerhuis *et al*.^1^ reported improved accuracy of sexing bovine embryos by using a cell surface histocompatibility-Y (H-Y) antigen, this methodology was not repeatable^2^. Subsequent research has used embryo biopsy^3^ and polymerase chain reaction (PCR)-based assays of sex chromosome-linked genes^4,5^, but these methods are generally labour intensive and error prone and may even damage normal embryonic development^2,6^.

A non-invasive genetic marker has also been reported to allow pre-selection of the sex of progeny^7^: a D4/XEGFP transgenic mouse line harbouring an X-linked *GFP* was generated by embryonic stem (ES) cell-mediated transgenesis in this study, but the incorporation of the *GFP* gene was random. Furthermore, X chromosome inactivation in mammals during embryologic development^8–11^ could terminate the expression of the X-linked transgene^7^. As one type of non-invasive genetic reporter, fluorescence proteins have been applied in many fields of research, such as identifying the genotype of transgenic animals^12–14^, tracing a specific cell lineage^15,16^, and studying the expression status of genes in time and space^7,17,18^. Therefore, non-invasive genetic reporters inspired us to consider how to use a non-invasive label to sex mammalian embryos.

Since the Y chromosome determines the male sex^19^, only male progeny can inherit Y chromosome-linked genetic markers. A ubiquitously expressed sex chromosome-linked reporter gene could be used to indicate the presence of an X or Y chromosome in the mouse embryo. Therefore, the expression of this reporter gene would reflect the sex of the progeny. Ultimately, a Y-linked transgenic mouse line could more stably transmit a genetic marker to single-sex progeny, and the presence of a labelled marker in the Y chromosome enables visual detection of the sex of embryos and animals.

The site-specific integration of exogenous genes can be readily achieved using CRISPR (clustered regularly interspaced short palindromic repeats)/Cas9^20–22^, and the type II bacterial CRISPR/Cas9 system has recently been engineered into an efficient genome-editing tool consisting of the Cas9 nuclease and a single guide RNA (sgRNA). The CRISPR/Cas9 system causes a double-strand break (DSB) at a specific gene locus to inactivate genes and has been used in many organisms^23–25^. Precise gene editing can be achieved by the homology-directed repair (HDR) mechanism^25–27^ when a donor DNA template is provided.

Based on the approach described above, we now demonstrate for the first time that the use of the XY^eGFP^ mouse line can help to distinguish between males and females. The ubiquitous expression of enhanced green fluorescence protein in males can be directly linked to sex and is suitable for routine embryo sex identification in mammals.

## Materials and Methods

### Mice

C57BL/6J and ICR mice were raised under specific pathogen-free (SPF) conditions. All animal experimental protocols were performed in accordance with the relevant ethical guidelines and regulations. All animal procedures used in this study were carried out in accordance with the Guide for the Care and Use of Laboratory Animals (8th edition, released by the National Research Council, USA) and were approved by the Institutional Animal Care and Use Committee (IACUC) of Guangxi University.

### Construction of plasmids

The donor vector contained a 0.75-kb left homologous arm (HA), a CAG-eGFP-bGH-PolyA cassette, and a 0.78-kb right HA. The backbone of the donor was pMD-19T (Takara, Kusatsu, Japan), and bGH-PolyA was amplified by PCR from px330 (Addgene, Watertown, MA, USA) and then inserted into the EcoRI/HindIII-digested pMD-19T vector using the ClonExpress II One Step Cloning Kit (Vazyme, Nanjing, China). To add a number of cloning sites in the vector, oligos were synthesized and purified through polyacrylamide gel electrophoresis (PAGE) (GENEWIZ, SuZhou, China) and subcloned into pMD-19T-bGH at the EcoRV restriction sites. The CAG-EGFP cassette was produced by digesting the plasmid CAG-EGFP-IRES-CRE (Addgene, Watertown, MA, USA) using SalI and NotI. Then, the CAG-EGFP cassette was inserted into the plasmid of pMD-19T-bGH-Oligos linearized by SalI and NotI to produce the vector pMD-19T-CAG-EGFP-bGH. The sequences of LHR and RHR were amplified by PCR from the genome and were subcloned into pMD-19T-CAG-EGFP-bGH at the 5′ KpnI/ClaI and 3′ BbsI/EcoRI restriction sites to complete the donor vector. The primer sequences used for PCR of bGH-PolyA, the 5′ and 3′ HAs, and the oligos are shown in Supplementary Table S2. All of the restriction enzymes were purchased from New England Biolabs (Ipswich, MA, USA). All of the enzymes used to amplify DNA in the construction of the donor were in 2 × Phanta Max Master Mix (Vazyme, Nanjing, China). PCR was performed with an initial denaturation at 95 °C for 3 min; 35 cycles of 95 °C for 15 s, 60 °C for 15 s and 72 °C for 40 s; and a final extension at 72 °C for 5 min.

### Production of Cas9 mRNA and sgRNA

The T7 promoter was added to Cas9 and sgRNA by PCR amplification of px260 (Addgene, Watertown, MA, USA) and px330 (Addgene, Watertown, MA, USA) using the primers listed in Table 1. The T7-Cas9 PCR product was purified and used as the template for *in vitro* transcription (IVT) using the mMESSAGE mMACHINE T7 Ultra Kit (Ambion, Austin, Texas, USA), and the T7-sgRNA PCR product was purified and used as the template for IVT using the MEGAshortscript^TM^ High Transcription Kit (Ambion, Austin, Texas, USA) according to the manufacturer’s instructions. Both *Cas9* mRNA and sgRNA were purified using the MEGA Clear Kit (Ambion, Austin, Texas, USA) according to the manufacturer’s instructions and eluted in RNase-free water.

**Table 1:** sgRNA sequence and the primers used to generate the templates for gRNA and Cas9 *in vitro* transcription.

Primer	Sequence(5′-3′)
sgRNA	AGACTAGAGAGGCTCAATTCTGG
sgRNA-IVT-F	TAATACGACTCACTATAGGGAGACTAGAGAGGCTCAATTCG TTTTAGAGCTAGAAATAG
sgRNA-IVT-R	AAAAGCACCGACTCGGTGCC
CAS9 IVT F	TAATACGACTCACTATAGGGAGATTTCAGGTTGGACCGGTG
CAS9 IVT R	GACGTCAGCGTTCGAATTGC

### Zygote microinjection, embryo culture and transplantation

Zygote microinjection was performed as previously described^28^. Briefly, mouse zygotes were obtained by mating super-ovulated female C57BL/6J mice (4 weeks old) with C57BL/6J male mice, and fertilized eggs were collected from their oviducts. *Cas9* mRNA (100 ng/µl), sgRNA (50 ng/µl) and the donor vector (100 ng/µl) were mixed and injected into the cytoplasm of zygotes with well-defined pronuclei in a droplet of HEPES-CZB (Millipore, Billerica, MA, USA) medium containing 5 µg/ml cytochalasin B (Sigma, ST. Louis, MO, USA) using a FemtoJet micro-injector (Eppendorf, Hamburg, Germany) with constant flow settings. The injected zygotes were cultured in KSOM medium with amino acids (Millipore, Billerica, MA, USA) at 37 °C under 5% CO_2_ in air until the blastocyst stage at 3.5 days. Then, 15–25 blastocysts were transferred into the uterus of pseudo-pregnant ICR females at 2.5 dpc as described previously^24^.

### Blastocyst genotyping analysis

The collection and transfer of single blastocysts were performed using a glass capillary under a dissection microscope as described in Zuo *et al*.^29^. A single blastocyst was picked based on its fluorescence and directly transferred into a PCR tube containing 2 µl of lysis buffer (0.1% Tween-20, 0.1% TritonX-100 and 4 µg/ml proteinase K). The samples were incubated for 20 min at 56 °C, and proteinase K was inactivated by heating at 95 °C for 10 min. PCR amplification was performed using nested primer sets (Table 2). Ex-Taq (Takara, Kusatsu, Japan) was activated at 95 °C for 3 min, and primary PCR was performed for 30 cycles at 95 °C for 30 s, 60 °C for 30 s and 72 °C for 1 min, with a final extension at 72 °C for 5 min. Secondary PCR was performed using 1 µl of the primary PCR product and a nested inner primer. PCR was carried out in the same reaction mixture, and the product was then gel purified and sequenced.

**Table 2:** Primers used for sex identification in embryos.

Primer	Sequence(5′-3′)	Products
Tyr OF	GTTATCCTCACACTACTTCTG	807 bp
Tyr OR	GTAATCCTACCAAGAGTCTCA	
Tyr IF	TCCTCACACTACTTCTGATG	788 bp
Tyr IR	GTCTCAAGATGGAAGATCAC	
Kdm5d OF	GCAGGCTACACAGGAGTA	491 bp
Kdm5d OR	AGGGACAGTAACAGGCATA	
Kdm5d IF	TTGGTGAGATGGCTGACT	457 bp
Kdm5d IR	GGACAGTAACAGGCATATGA	

### Detection of insertion mutations in mice and off-target analyses

Mouse tail DNA was purified using a TIANamp Genomic DNA Kit (TIANGEN, China) according to the manufacturer’s instructions. The integration site of the CAG-EGFP cassette was detected by primers located separately in the mouse genome and at the CAG-eGFP-bGH cassette in 5′ and 3′ junctions. The fragment length of the 5′ junction was amplified by placing the forward primer in the mouse genome and the reverse primer in the CAG promoter. The 3′ junction was detected by placing the forward primer in the polyA signal and the reverse primer in the mouse genome. The primer target in the genome flanks the HA to exclude interference from the original plasmid. The primer sequences used for PCR are listed in Table 3. The enzymes used for PCR were from Vazyme (Nanjing, China), and PCR was performed as follows: initial denaturation at 95 °C for 3 min; 35 cycles of 95 °C for 15 s, 60 °C for 15 s, and 72 °C for 40 s; and a final extension at 72 °C for 5 min. The products were stored at 4 °C for evaluation via agarose gel electrophoresis.

**Table 3:** Primers used for the 5′ and 3′ junctions.

Locus	Sequence (5′-3′)	products
5′F	ACCGTAAATACTCCACCC	1172 bp
5′R	GTCTGAAGACAGCTACAG	
3′F	CTGCTGCCCGACAACCACT	1016 bp
3′R	ACCAGAAGAGGGCATCAGAT	

As previously reported^30^, potential off-target sites of sgRNA were predicted using online software (http://www.rgenome.net/cas-offinder/). We collected all the off-target sites that had no more than 3 mismatches for the sgRNA and then broadened the search regions to 250 base pairs up- and downstream of the potential off-target sites. For amplification of the loci, PCR amplifications were performed as follows: initial denaturation at 95 °C for 3 min; 35 cycles of 95 °C for 30 s, 60 °C for 30 s, and 72 °C for 1 min; and a final extension at 72 °C for 5 min. The products were then stored at 4 °C, and the primers used are listed in Table 4.

**Table 4:** Primers used for off-target analysis.

Locus	Sequence (5′-3′)	products
Off-target 1-F	AAGAGTAGCCGAGCAGTG	516 bp
Off-target 1-R	GACTCAAATAATAAGTGGG	
Off-target 2-F	CCAAGACCTTGTCGCTGAC	546 bp
Off-target 2-R	TGCCCACCTCCTTCCTAT	
Off-target 3-F	GGGGTTGAGTTTGGCTTTC	702 bp
Off-target 3-R	TGGGATATGGGAGGGTTT	
Off-target 4-F	GGCTACGGTACATCACTA	493 bp
Off-target 4-R	TCAGACCAGAGTCCAAGT	
Off-target 5-F	CCCTCTTCTGGAGTGTCT	487 bp
Off-target 5-R	TGAACCTTGCTCTGCCTA	
Off-target 6-F	CTGGTCCTAACAGGTGCT	619 bp
Off-target 6-R	GGAGTAAAGTTGCAGGTGA	
Off-target 7-F	ACCACCATCACCCTCAGT	491 bp
Off-target 7-R	TAAGAGCCCGAGACAATC	
Off-target 8-F	GCTTTAGAAGAAGGGACG	455 bp
Off-target 8-R	AAGAGGGAGACACTGATAGA	
Off-target 9-F	AGCTGGCACAGTGAAGAA	537 bp
Off-target 9-R	GCTTGTCTGGGACTATACCT	

### Statistical analysis

Data are presented as the mean ± SEM. Statistical analysis was performed using Prism 7 (GraphPad, San Diego, CA, USA), and significant differences were determined using Student’s unpaired t test. P > 0.05 was considered not significantly different.

## Results

### Strategy for the generation of Y-Chr-eGFP mice with the CAG promoter

**Figure 1:**
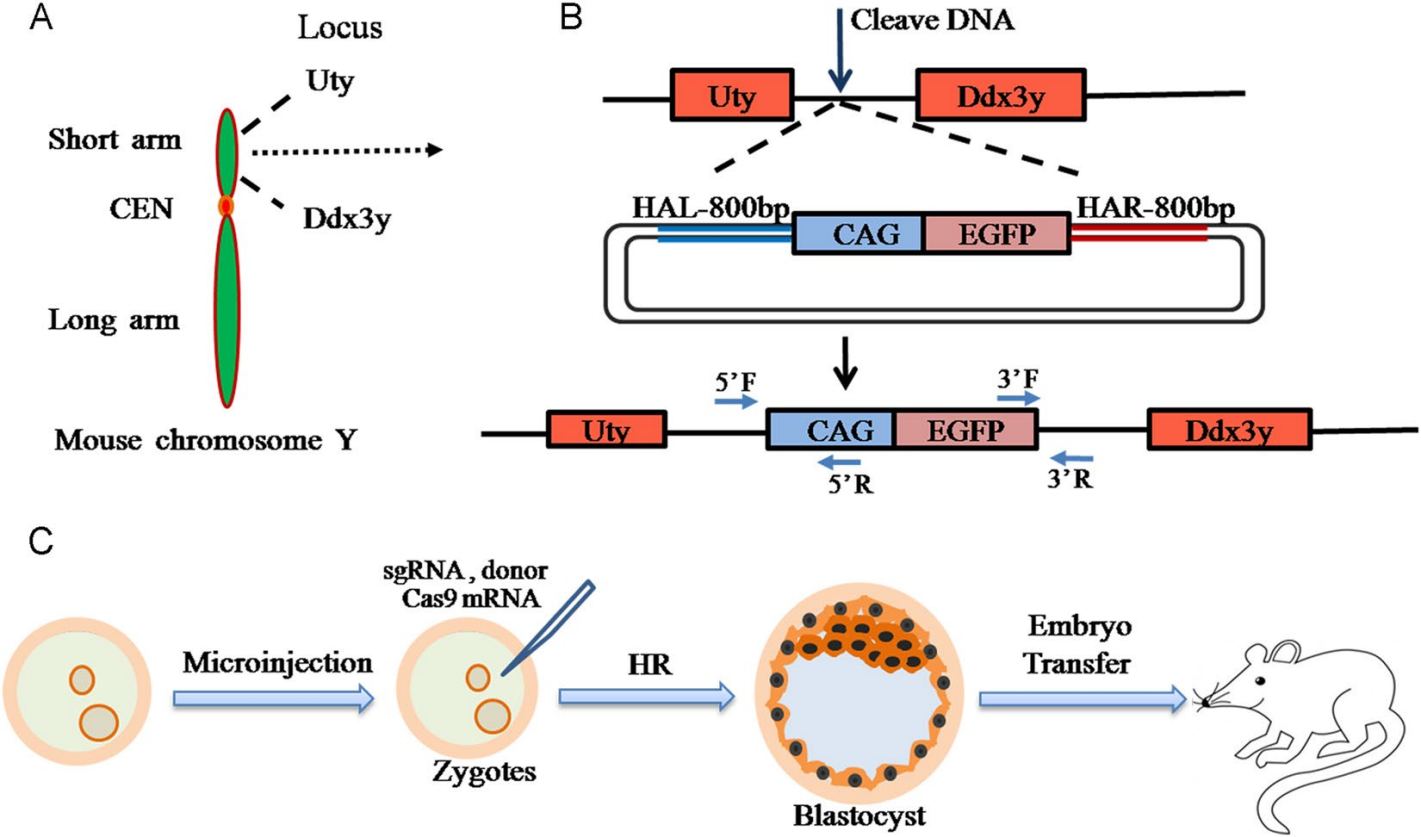
Strategy for the generation of Y-Chr-eGFP mice with a CAG promoter. (**A**) Targeted locus in the Y chromosome: intergenic region sequence of the *Ddx3y* and *Uty* genes, which are both located on the short arm of the Y chromosome. (**B**) Schematic overview of the homologous independent DSB repair pathway at the target locus. (**C**) Flowchart for the generation of the gene-edited mice.

A specified locus for the integration of *eGFP* was selected in the intergenic region of the two contiguous genes *Ddx3y* and *Uty* of the Y chromosome short arm (Fig. 1A). Our experimental design is based on that described by Chu *et al*.^31^ and involves the co-injection of *Cas9* mRNA, sgRNA, and a donor plasmid. The donor plasmid contains an 800 base pair homology sequence upstream and downstream of the insertion cassette. Cas9 is guided by the sgRNA and results in the incorporation of the donor plasmid via homology-dependent repair (Fig. 1B). The reason for employing a cytomegalovirus early enhancer element and chicken β-actin (CAG) is that it is a strong promoter and can enhance the expression level of the transgene^32^. Fertilized eggs were collected from oviducts by crossing super-ovulated female C57BL/6J mice with C57BL/6J males, and Y-Chr-eGFP mice were produced from zygotes by microinjection and embryo transfer (Fig. 1C).

### Generation of Y-Chr-eGFP transgenic mice

**Figure 2:**
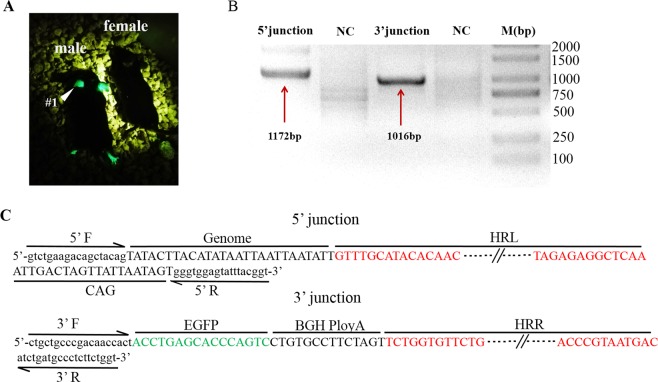
Generation of Y-Chr-eGFP mice. (**A**) Photograph of Y-Chr-eGFP mouse #1 and the control male mouse. (**B**) Genotyping analysis of the Y-Chr-eGFP mice: PCR products amplified from the 5′ and 3′ junction sites of DNA samples from Y-Chr-eGFP mouse #1. NC, negative control from WT male mice. M, DNA marker. (**C**) Sequencing results of the integration sites in Y-Chr-eGFP mice. DNA sequencing of the PCR products amplified from the 5′ and 3′ junction sites of DNA samples from Y-Chr-eGFP mouse #1.

We first generated Y-Chr-eGFP transgenic mice through CRISPR/Cas9-mediated homology-dependent repair. Whole fluorescent mice could be identified when the GFP-positive founder was exposed to ultraviolet light, as shown in Fig. 2A. We produced germline-chimeric mice, and then Y-Chr-eGFP mice were produced through test-cross analysis with wild-type (WT) female mice. To verify the accuracy of the integration site of the *GFP* gene in the F1 generation of Y-Chr-eGFP mice, genotyping analysis was performed by PCR. The amplified bands (Fig. 2B) of the 5′ and 3′ junctions had sizes that were consistent with expectations, and their exact sizes were determined by sequencing (Fig. 2C). The results showed that the length of the fragment was 1172 base pairs for the 5′ junction and 1016 base pairs for the 3′ junction. Furthermore, these results indicate that the *GFP* gene was successfully inserted into the specific sites.

### Off-target analysis of Y-Chr-eGFP mice

**Figure 3:**
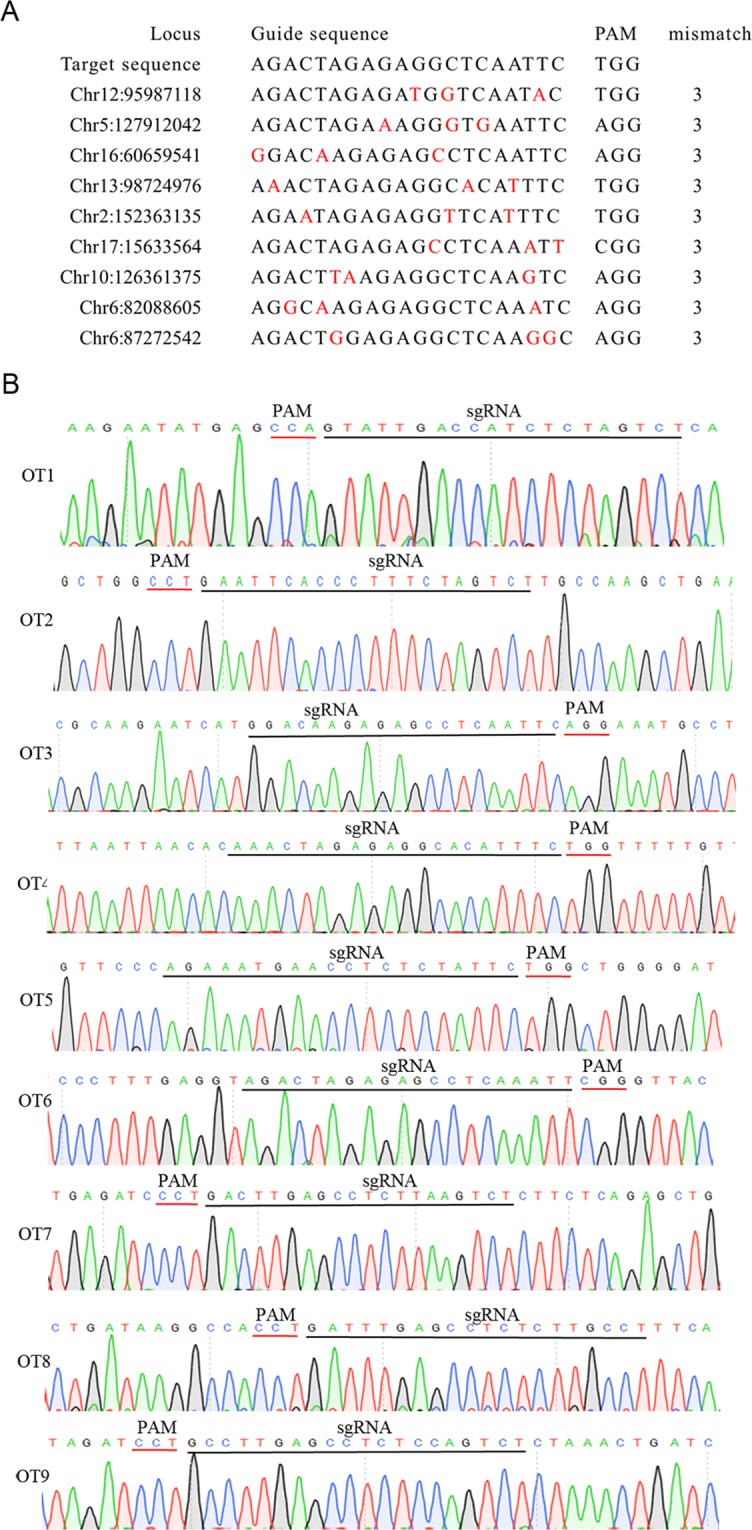
Off-target analyses in the Y-Chr-eGFP mice. (**A**) Off-target sites resulting from sgRNA targeting were predicted using online software. Mismatches for up to 10 potential off-target sites were selected for analysis. Red indicates a mismatch with the targeted sequence. (**B**) Sequencing results of the off-target sites in Y-Chr-eGFP mice. DNA sequencing of the PCR products amplified from these genomic sites were TA cloned and sequenced.

To assess the off-target effects of the CRISPR/Cas9 system in Y-Chr-eGFP mice, we examined 9 off-target sites for the sgRNA used in Y-Chr-eGFP mice (Fig. 3A). DNA sequencing of the PCR products that covered the potential off-target loci showed that no mutations occurred (Fig. 3B).

### Sex identification of embryos from (Y-Chr-eGFP♂ x WT♀)

**Figure 4:**
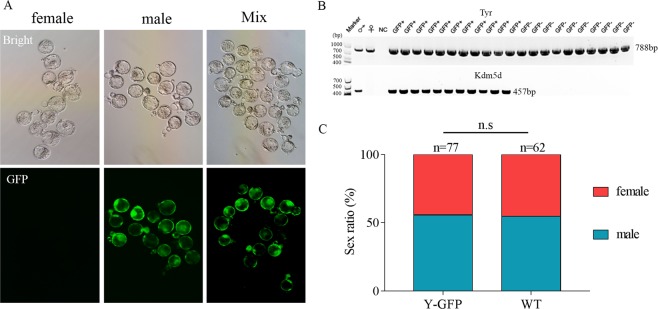
Sex identification of embryos. (**A**) Representative fluorescence images of embryos. Mixed zygotes cultured for 3.5 days were collected by crossing a Y-Chr-eGFP male mouse with a WT female mouse. Female, GFP-negative embryos under a fluorescence microscope; male, GFP-positive embryos under a fluorescence microscope. (**B**) Electrophoresis results of PCR products for sexing GFP+ and GFP− embryos. The Tyr gene is located on the autosome and serves as a control. The Kdm5d gene is located on the Y chromosome and serves as a male-specific control. (**C**) Sex ratio of embryos obtained from mating superovulation females with Y-Chr-eGFP male mice and WT male mice. There was no significant difference between Y-Chr-eGFP mice and WT male mice. “n” is the sample size of numbered embryos.

We produced germline-chimeric mice, and then Y-Chr-eGFP mice were produced through test-cross analysis with WT female mice. The expression of green fluorescent protein can occur only in male offspring of the F1 generation (Fig. S4). Thus, the GFP-positive founder is a germline chimaera, and the eGFP gene of the Y-Chr-eGFP founder can be stably transmitted to the male offspring by germline transmission. Then, we collected the zygotes by mating Y-Chr-eGFP transgenic male mice (F1) to WT females. Green fluorescence was observed in half of the blastocysts that developed from the zygotes (Fig. 4A); the expression of green fluorescent protein in the offspring showed the stable germline transmission of the *eGFP* gene. Green blastocysts and non-green blastocysts were easily observed and separated under a fluorescence microscope when there was high expression of the *eGFP* gene. To evaluate the concordance between the reporter gene and PCR-based assays for sexing, 21 blastocysts were subjected to PCR analysis. Tyrosinase (*Tyr*) (Gene ID: 22173) is a key enzyme in melanin biosynthesis that is located on chromosome 7 and can serve as a positive control; lysine (K)-specific demethylase 5D (*Kdm5d*) (Gene ID: 20592) is located on the Y chromosome and can be used to identify males. As shown in Fig. 4B, a positive band for *Tyr* was observed at the 788 base pair position in all 21 embryos, but a positive band for *Kdm5d* was observed at the 457 base pair position only in the 11 green embryos (Fig. 4B). Furthermore, when the GFP-positive and GFP-negative blastocysts were implanted into foster mothers, the offspring formed from GFP-positive embryos were all males, and those formed from the other embryos were all females. All of these results reflect that the Y-Chr-eGFP founder is a germline chimaera, and the *eGFP* gene of the Y-Chr-eGFP founder can be stably transmitted to the male offspring by germline transmission. To test whether carrying the *GFP* gene in the Y chromosome impacted the development ability of XY^eGFP^ embryos, we separately mated Y-Chr-eGFP mice and WT male mice with super-ovulated female mice. Then, zygotes were collected and cultured *in vitro*, and 3.5 days later, all embryos were subjected to PCR to identify their sex. The results showed no difference in the sex ratio of males compared with WT (Fig. 4C).

## Discussion

The ability to produce offspring of the desired sex to take advantage of the different values of males and females is a significant benefit for livestock production, and several techniques have been developed to determine the sex of pre-implantation embryos in previous research. These techniques include embryo karyotyping^33^, fluorescence *in situ* hybridization (FISH) based on a sex chromosome-specific DNA probe^34^, and PCR-based assays^35–37^, but the sexing efficiencies of these techniques are closely related to the size of the embryo biopsy and may result in decreased pregnancy rates following the transfer of desmi-embryos^38,39^.

The CRISPR/Cas9 system has been used to produce gene-edited animals by the microinjection of *Cas9* mRNA, sgRNA, and a donor template into the cytoplasm of zygotes, which greatly shortens the amount of time required for producing transgenic animals. In our current study, the XY^*eGFP*^ mouse line was produced to enable alteration of the sex ratio in pregnancy. As shown in Hadjantonakis *et al*.^7^, labelled X chromosomes can also be used to sex pre-implantation embryos; however, it is difficult to define the sex after X chromosome inactivation during embryologic development. Furthermore, X-linked GFP male mice can pass their labelled X chromosome only to their female offspring. The X^*GFP*^Y mutant male mouse line is difficult to maintain by only crossing with X^*GFP*^X or X^*GFP*^X^*GFP*^ female mice in accordance with Mendelian inheritance. Therefore, X^*GFP*^X or X^*GFP*^X^*GFP*^ female mice are needed to breed X-Chr-eGFP male mice. However, Y-linked GFP can exclusively be inherited by male offspring, and all of the resulting male genotypes are XY^*eGFP*^. The first report on Y chromosome tracing by a labelled gene was by Yamamoto *et al*., who demonstrated that a marked Y chromosome was helpful for monitoring the presence of the Y chromosome in Tg mouse-derived ES cell lines^40^. Their research is a great inspiration for us. However, there are many differences between our study and that of Yamamota *et al*. First, the purpose of our study is different. The main purpose of Yamamota *et al*. was to monitor the presence of the Y chromosome in Tg mouse-derived ES cell lines, while our purpose was to control the sex ratio of mammals by sexing pre-implantation embryos. Second, we used a different method to produce gene-modified mice. The CRISPR-mediated integration of exogenous DNA is more efficient than traditional approaches. We summarize the knock-in efficiency in offspring in Supplementary Table S1. Only one positive founder was identified, and the efficiency of the HR donor could reach 2.4%. As shown in Yao *et al*., the knock-in efficiency was no greater than 5% in blastocysts at different target sites^25^. As reported by Wang *et al*., the efficiency of CRISPR/HDR is in the range of 0.5–20%^41^. The HDR-mediated knock-in efficiency is still very low efficiency in animal embryos and tissues *in vivo*^42,43^, which is closely related to the length of the HA, the insertion fragment, and the target sites. At present, many studies have been conducted to improve the knock-in efficiency, such as using an inhibitor of the NHEJ pathway^43^, and optimizing different strategies based on targeted integration^22,25^, but it is still relatively low. The factors affecting knock-in efficiency remain to be further investigated to facilitate the widespread use of gene editing tools. However, it is still far higher than that of the traditional method (10^−6^~10^−9^)^44^. Third, the choice of target locus was different. In our study, the *eGFP* gene was inserted into a pre-determined site in the intergenic region of the *Ddx3y* and *Uty* genes in the Y chromosome, which overcomes the defect of random integration, thus guaranteeing the accuracy and reliability of the method. The integration locus is approximately 5.5 kb from the *Uty* gene and 9.3 kb from the *Ddx3y* gene, as proposed by Zuo *et al*.^28,29^. All male mice with deletions of these two genes and their F1 male offspring were fertile and had normal reproductive ability. The mRNA expression levels of the *Ddx3y* and *Uty* genes in the MEFs derived from Y-Chr-eGFP and WT mice were identified by Q-PCR, and no significant differences were observed, as shown in supplementary Fig. S1. In addition, our method has no effect on the development of XY embryos *in vitro* compared with the results in WT embryos, and there was no skewing of the sex ratio, which indicates that our method can be widely used for sex selection in agricultural production.

Although low transgene expression levels may be unsuited for simple visual examination, as suggested by Cornett *et al*.^45^, this work shows that *GFP* works well in distinguishing labelled and non-labelled mouse embryos when under the control of a strong CAG promoter. As indicated in previous research^14^, no adverse effect was detected in living cells as a result of the expression of *eGFP*. The onset of transgene expression in embryos is dependent on its particular promoter^32^, and based on the research performed by Hadjantonakis *et al*.^7^, expression of an exogenous gene could be detected at the morula stage. As shown by the experimental results, Y-linked *eGFP* can be used for visual detection of the sex of pre-implantation embryos with 100% accuracy.

Brief irradiation is not detrimental to the viability of embryos^12,46^, and as shown in our research, early blastocysts exposed to brief irradiation under a fluorescence microscope successfully developed into pups after transfer to the uterus of pseudo-pregnant foster mothers. Therefore, green male and non-green female embryos were separated at the early blastocyst stage for specific embryo transfer to control the sex bias of the offspring. No off-target effects were detected in XY^*eGFP*^ mice produced by CRISPR/Cas9, which was consistent with previous studies that showed that off-target mutations are rare in gene-edited animals produced by the CRISPR/Cas9 technique^47^.

The procedures reported here demonstrate for the first time that a Y chromosome tracer can be used to sex mammalian pre-implantation embryos produced by the CRISPR/Cas9 system. This study also provides a rapid and non-invasive approach for identifying the sex of embryos without compromising the accuracy, feasibility and practicality of the method, and it will have to be modified for similar work in other species.

## Supplementary information

Identification of the Sex of Pre-implantation Mouse Embryos Using a Marked Y Chromosome and CRISPR/Cas9
