# Supplementary material for: Identification of the Sex of Pre-implantation Mouse Embryos Using a Marked Y Chromosome and CRISPR/Cas9
Source: Sci Rep. 2019 Oct 4;9:14315. doi: 10.1038/s41598-019-50731-x (PMC6778187; doi:10.1038/s41598-019-50731-x)
Supplement: Supplementary file 1 — Identification of the Sex of Pre-implantation Mouse Embryos Using a Marked Y Chromosome and CRISPR/Cas9 [file 41598_2019_50731_MOESM1_ESM.docx]

**Supplementary Information**

**Identification of the Sex of Pre-implantation Mouse Embryos Using a Marked Y Chromosome and CRISPR/Cas9**

Running title: Sex Determination in Pre-implantation Embryos

Xiuling Zhao ^1^, Wei Wei ^2^, Hong Pan ^1^, Junyu Nie ^1^, Dongrong Chen ^1^, Pengfei Zhang ^1^, Fumei Chen ^1^, Qiang Fu ^1^, Erwei Zuo ^3, *^, Yangqing Lu ^1, *^, Ming Zhang ^1, *^

^1^ State Key Laboratory for Conservation and Utilization of Subtropical Agro-Bioresources, Animal Reproduction Institute, Guangxi University, Nanning

530004, Guangxi, PR China

^2^ Shenzhen Hengsheng Hospital, Shenzhen 518101, PR China

^3^ Center for Research in Animal Genomics, Agricultural Genome Institute at

Shenzhen, Chinese Academy of Agricultural Sciences, Shenzhen 518124, PR China

* Corresponding authors. E-mail addresses: erweizuo@163.com (E.W. Zuo), luyangqing@126.com (Y.Q. Lu), mingzhang@gxu.edu.cn (M. Zhang).

**Contents:**

**Figure S1.** The mRNA expression level of *Ddx3y* and *Uty* gene in the MEF derived from Y-Chr-eGFP and WT mice

**Figure S2.** Sequencing results of 5’ junction

**Figure S3.** Sequencing results of 3’ junction

**Figure S4.** Photograph of Y-Chr-eGFP male mice (2 days after birth)

**Table S1.** Y-Chr-eGFP mice generated by HDR-mediated targeted integration

**Table S2.** Primer sequences used for construction of donor plasmid

Full-length gel picture of Fig. 2B

Full-length gel picture of Fig. 4B Upper gel

Full-length gel picture of Fig. 4B Lower gel


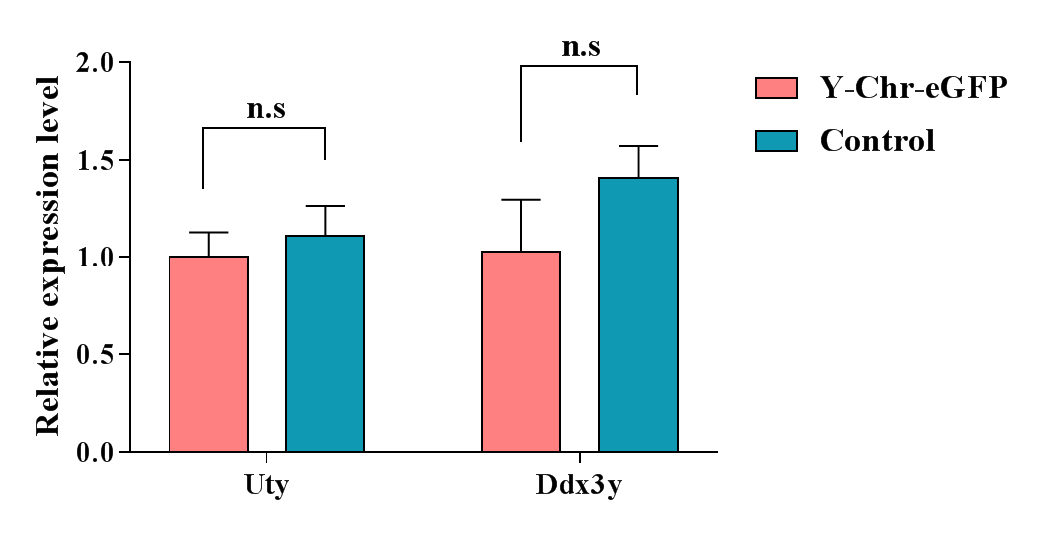


**Figure S1.** The mRNA expression level of *Ddx3y* and *Uty* gene in the MEF derived from Y-Chr-eGFP and WT mouse


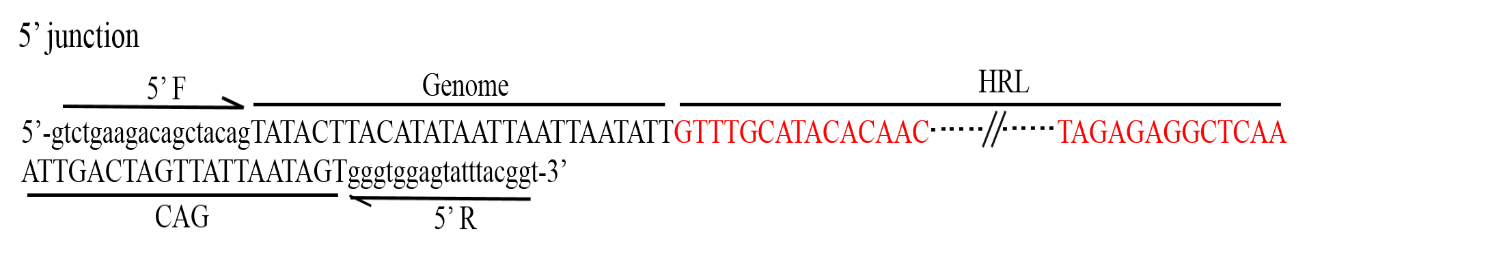

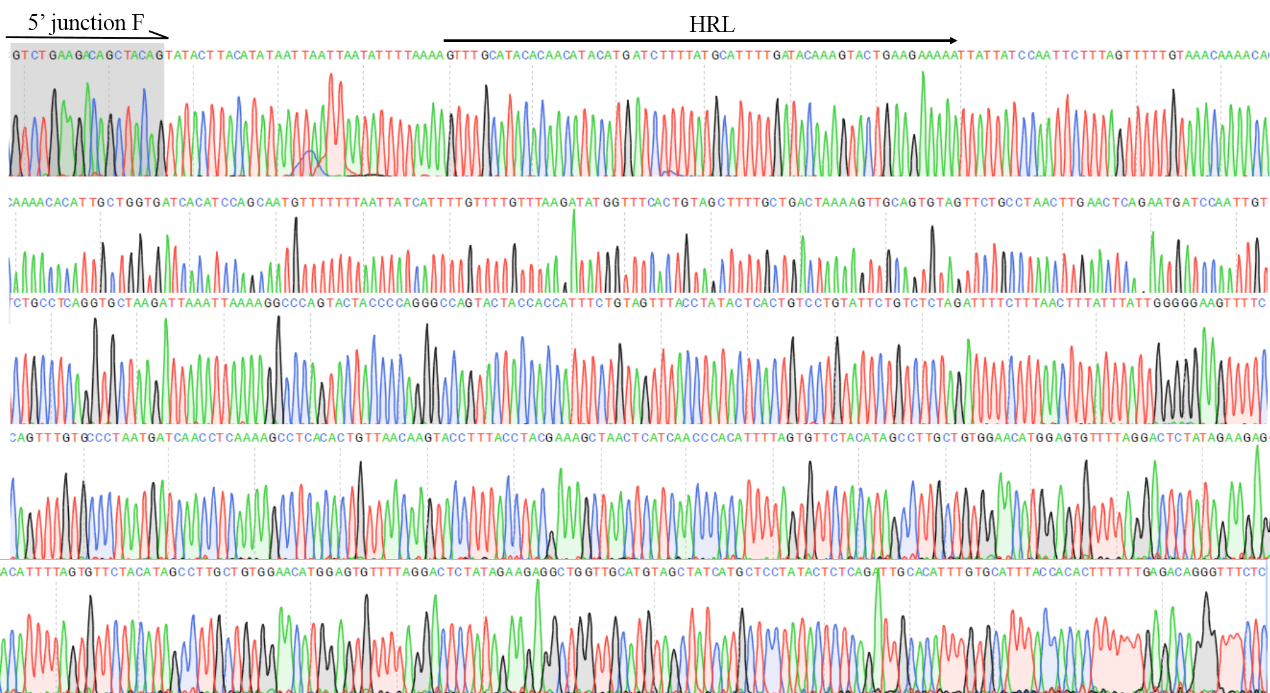

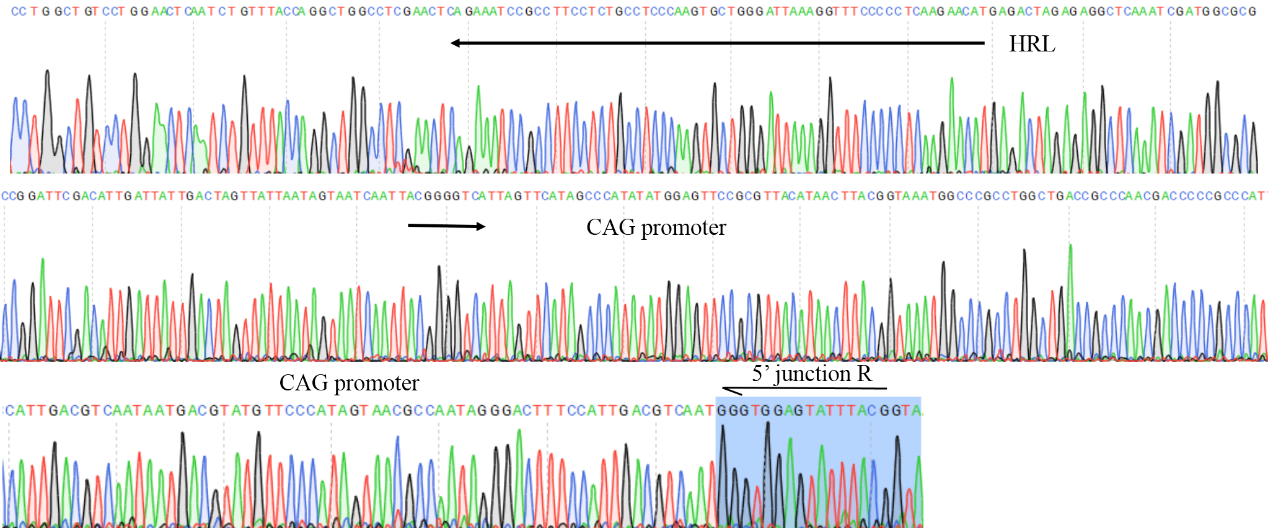


**Figure S2.** Sequencing results of 5’ junction


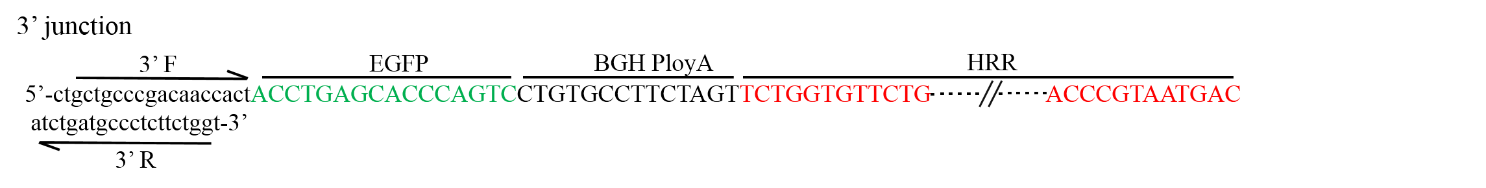


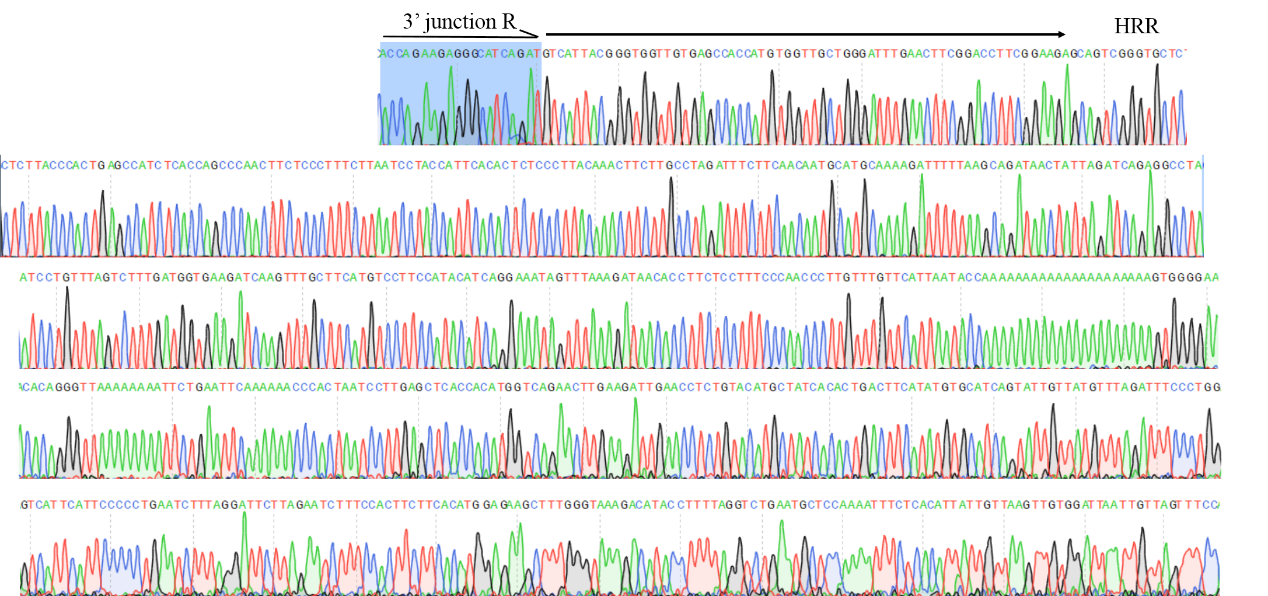

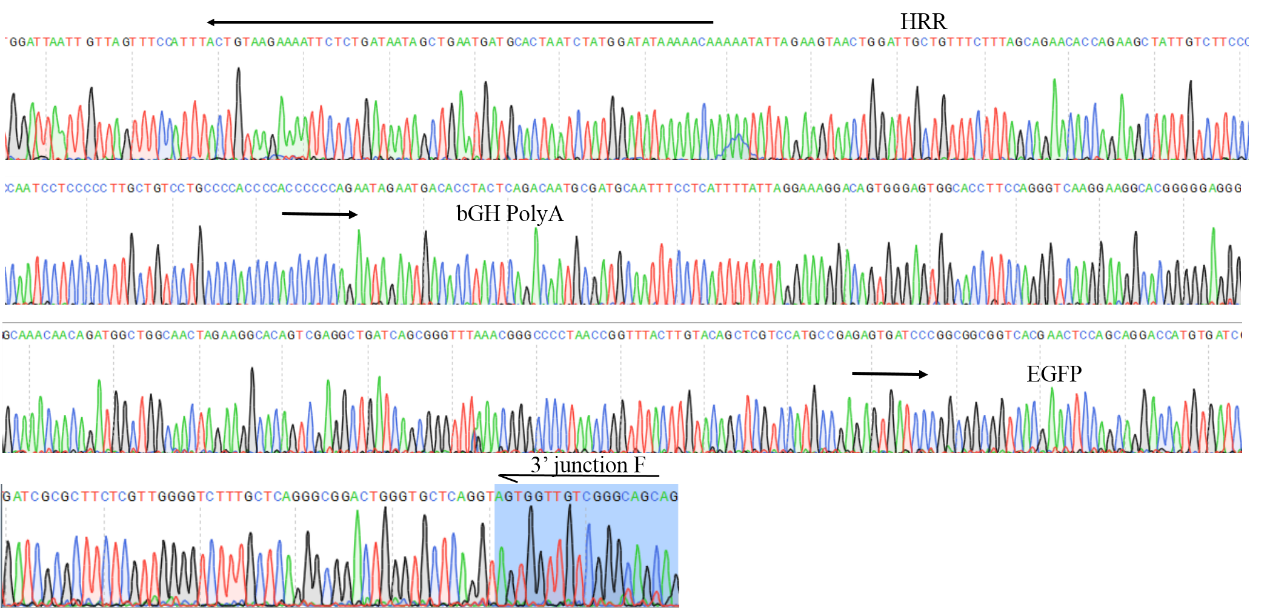


**Figure S3.** Sequencing results of 3’ junction


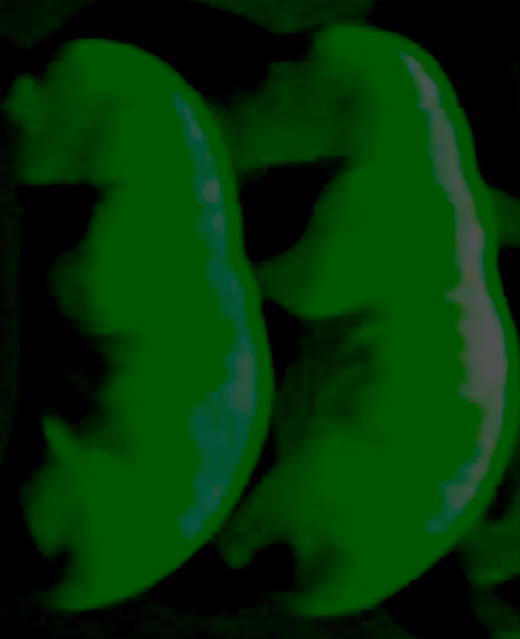


**Figure S4.** Photograph of Y-Chr-eGFP male mice (2 days after birth)
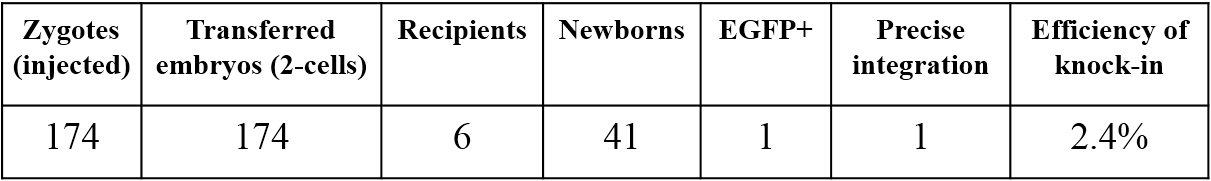


**Table S1.** Y-Chr-eGFP mice generated by HDR-mediated targeted integration


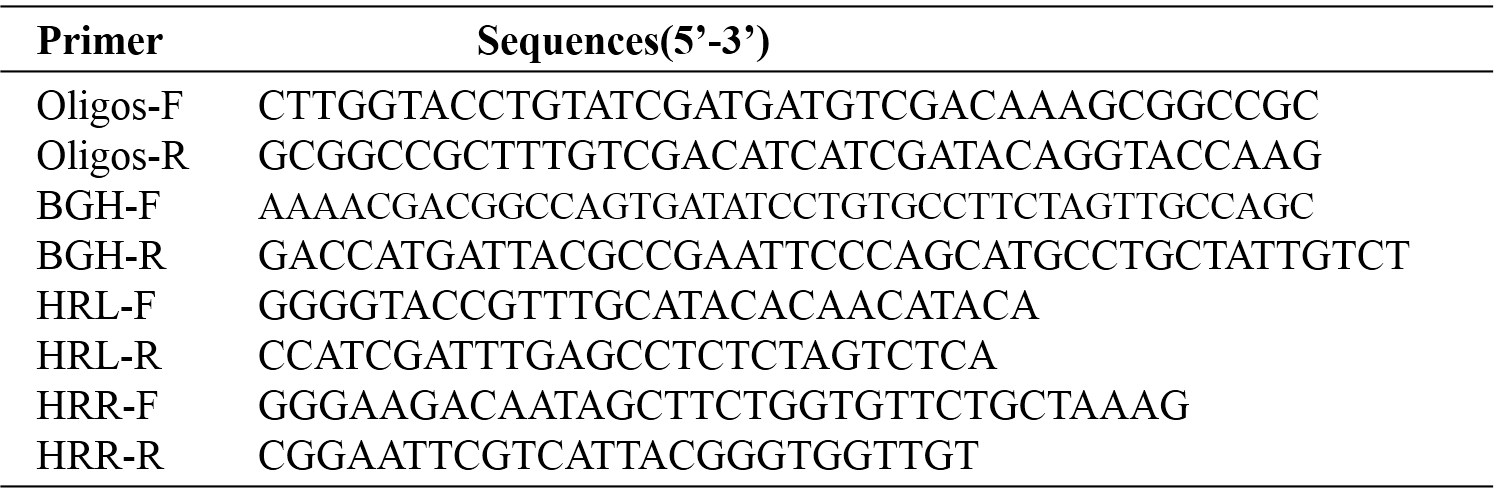
**Table S2.** Primer sequences used for construction of donor plasmid


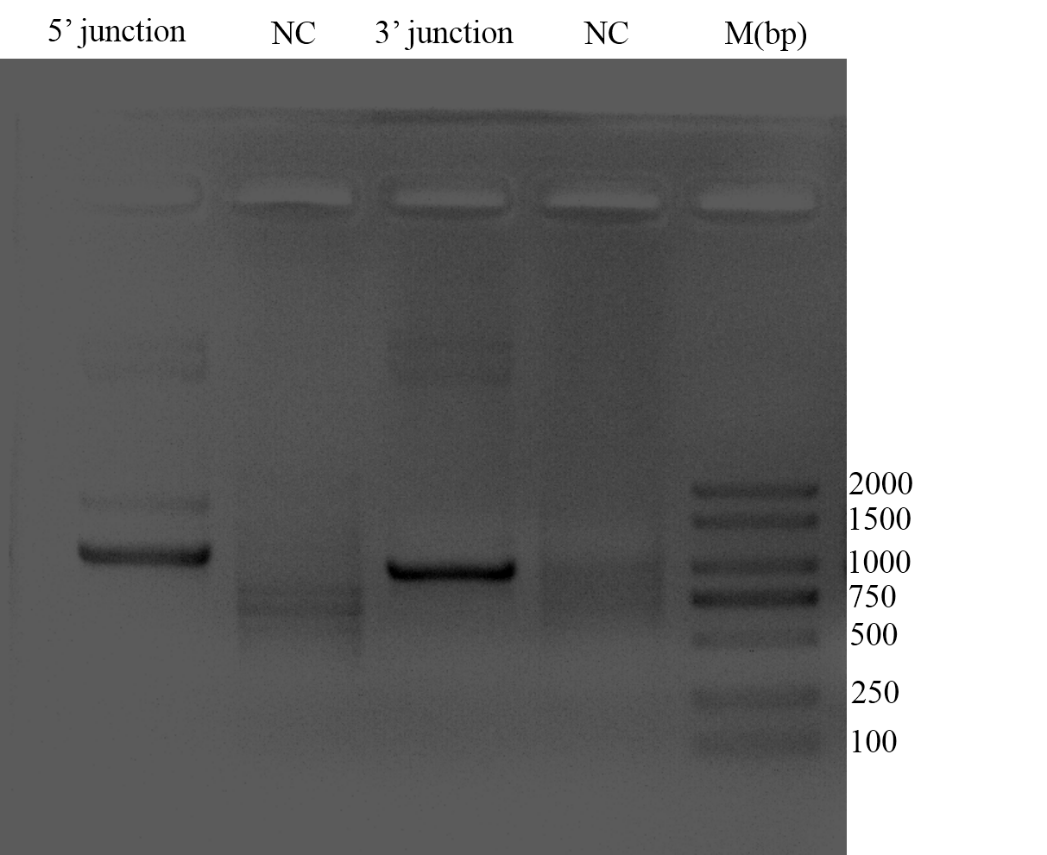


Full-length gel picture of Fig. 2B


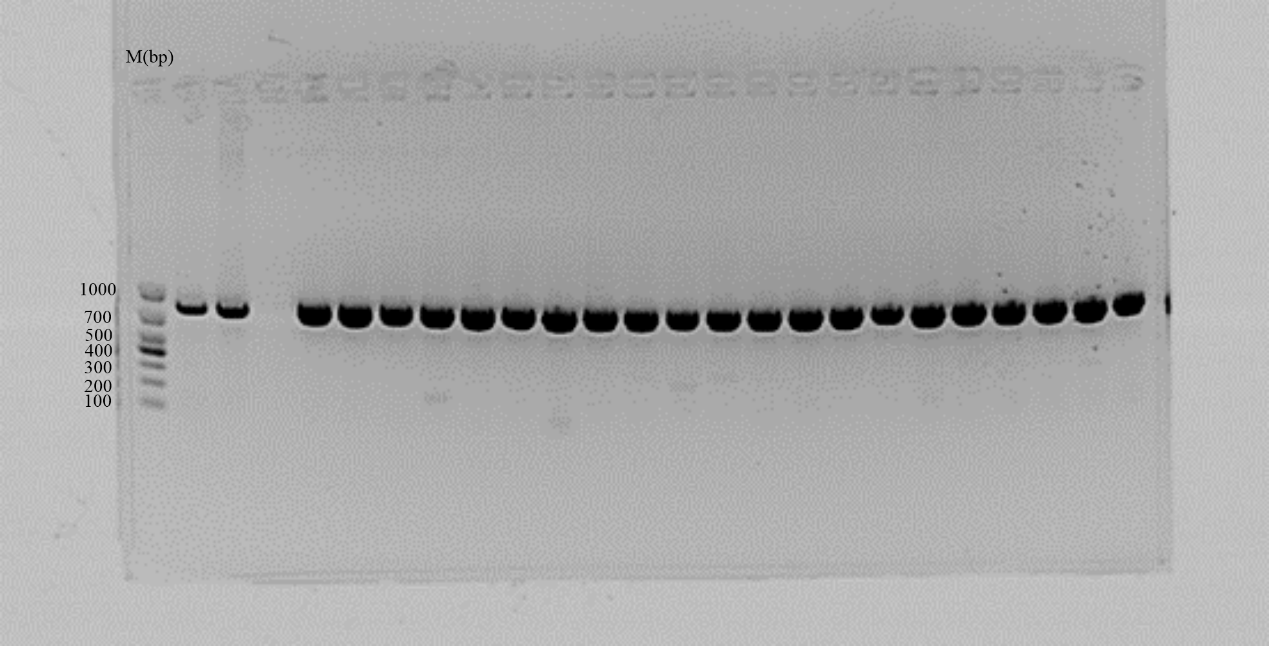


Full-length gel picture of Fig. 4B Upper gel


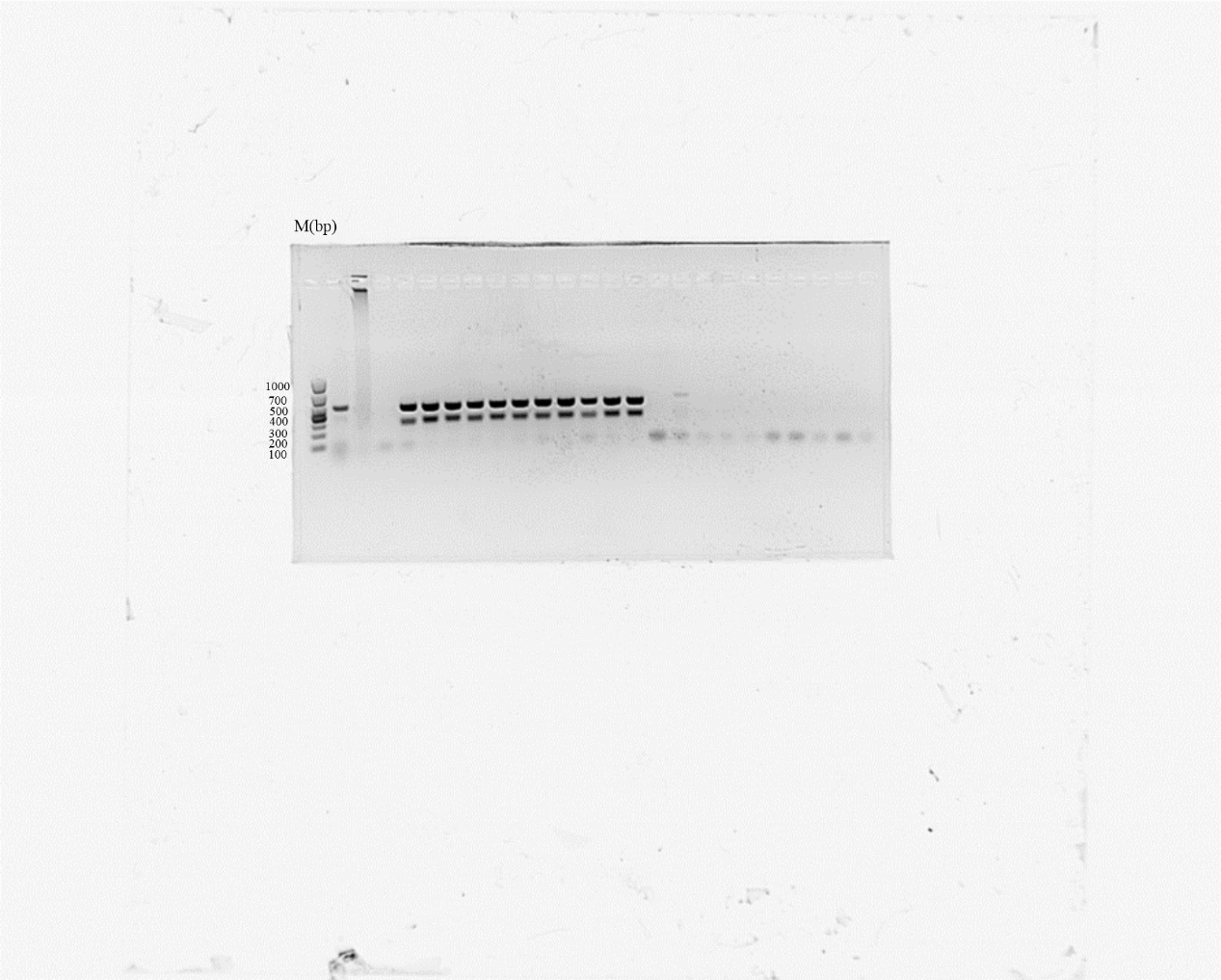


Full-length gel picture of Fig. 4B Lower gel
